# Cytoprotective Properties of a New Nanocomplex of Selenium with Taxifolin in the Cells of the Cerebral Cortex Exposed to Ischemia/Reoxygenation

**DOI:** 10.3390/pharmaceutics14112477

**Published:** 2022-11-16

**Authors:** Elena G. Varlamova, Venera V. Khabatova, Sergey V. Gudkov, Egor Y. Plotnikov, Egor A. Turovsky

**Affiliations:** 1 Institute of Cell Biophysics of the Russian Academy of Sciences, Federal Research Center “Pushchino Scientific Center for Biological Research of the Russian Academy of Sciences”, 142290 Pushchino, Russia; 2 Prokhorov General Physics Institute of the Russian Academy of Sciences, 38 Vavilove st., 119991 Moscow, Russia; 3 A.N. Belozersky Institute of Physico-Chemical Biology, Lomonosov Moscow State University, 119992 Moscow, Russia; 4 V.I. Kulakov National Medical Research Center of Obstetrics, Gynecology and Perinatology, 117997 Moscow, Russia

**Keywords:** taxifolin, ROS, oxygen–glucose deprivation, cell death, cortex, astrocyte, neurons, selenium nanoparticles, calcium, gene expression, nanocomplex

## Abstract

The neuroprotective effect of the natural antioxidant taxifolin (TAX) is well known for ischemic pathologies. However, the limitations of taxifolin application are described—poor solubility, low ability to penetrate the blood–brain barrier, and side effects from high doses for stroke therapy. We proposed the problem of targeted delivery of taxifolin and achievement effective concentrations could be solved by developing a nanocomplex of selenium nanoparticles (SeNPs) with taxifolin (Se–TAX). In this study, we developed a selenium–taxifolin nanocomplex based on selenium nanoparticles with a 100 nm size. It was shown that TAX, SeNPs, and Se–TAX were all able to suppress the production of ROS in neurons and astrocytes under exposure to exogenous H_2_O_2_ and ischemia-like conditions. However, the Se–TAX nanocomplex appeared to be the most effective, displaying a lower working concentration range and negligible pro-oxidant effect compared with pure SeNPs. The mechanism of Se–TAX beneficial effects involved the activation of some antioxidant enzymes and the suppression of ROS-generating systems during OGD/reoxygenation, while TAX and “naked” SeNPs were less effective in regulating the cellular redox status. Naked SeNPs inhibited a global increase in Ca^2+^ ions in cytosol, but not OGD-induced hyperexcitation of the neuroglial network, while Se–TAX suppressed both [Ca^2+^]i rise and hyperexcitation. The effect of TAX at similar doses appeared exclusively in inhibiting OGD-induced hyperexcitation. Analysis of necrosis and apoptosis after OGD/reoxygenation revealed the highest efficiency of the Se–TAX nanocomplex as well. Se–TAX suppressed the expression of proinflammatory and proapoptotic proteins with simultaneous activation of protective genes. We conclude that the Se–TAX nanocomplex combines the antioxidative features taxifolin and the antiapoptotic effect of nanoselenium, involving the regulation of Ca^2+^ dynamics.

## 1. Introduction

Ischemic stroke, resulting from various blood flow disorders, affects millions of people worldwide. Stroke therapies are significantly limited due to rapid brain metabolism and poor transport of most neuroprotectors across the blood–brain barrier [1]. The cascade of events that occurs during cerebral ischemia includes: a disruption in the supply of oxygen and nutrients to the tissue, an increase in the glutamate release and a violation of its utilization, an increase in the cytosolic Ca^2+^ ([Ca^2+^]_i_) concentration in neurons and astrocytes, a disruption in the mitochondrial functioning, and most importantly, an overproduction of reactive oxygen species (ROS).

All these processes lead to excitotoxicity, hyperexcitation of neuronal networks, necrosis, and apoptosis activation [2,3]. The search for effective ways to prevent and treat stroke is still one of the urgent tasks in biology and medicine. Therefore, neuroprotective compounds with antioxidant and anti-inflammatory properties are of utmost interest. However, along with gaps in the mechanisms of action of most active compounds, there is a problem of high doses of utilization and their side effects on healthy cells. Moreover it is necessary to develop a system for the rapid targeted delivery of antioxidants to the area of brain damage.

Taxifolin (quercetin, TAX) is an important bioflavonoid polyphenolic antioxidant, and it can be found in fairly large amounts in fruits, vegetable oils, red wine, tea, Siberian larch, and so on [4,5]. Taxifolin is approved by the U.S. Food and Drug Administration (FDA) and exhibits numerous beneficial effects, including antioxidant, anti-inflammatory, anticancer, cardioprotective, and neuroprotective effects [6,7]. The properties of taxifolin as a powerful and effective antioxidant have been known for a while [8,9]. Taxifolin demonstrates a rapid neuroprotection, primarily suppressing ROS production in the inhibitory population of GABA neurons. Additionally, taxifolin can influence gene expression regulating the balance between cell survival and death [10]. The clinical application of taxifolin and its derivatives has been significantly limited due to their poor bioavailability, resulting from low water solubility, extensive first-pass metabolism, and limited penetration through the blood–brain barrier [6,11].

The trace element selenium (Se) has a pleiotropic effect and a high therapeutic potential for the treatment of various diseases. It attracts a lot of attention for the use in therapy and nanomedicine. According to the World Health Organization (WHO), the Se consumption rate is: for adult women, 55 µg/day, for adult men, 70 µg/day. The upper permissible level of Se consumption ranges from 300 to 600 μg/day. The toxic dose is considered to be 900 μg/day [12,13,14]. Thus, the window between therapeutic and toxic doses is very narrow.

Selenium and its compounds’ pleiotropy appears, first of all, in the apoptotic mechanism induction in tumor cells [15,16,17] and, second of all, in the activation of cytoprotective signaling pathways in healthy cells with various injuries [18,19]. Selenium compounds have some antioxidant properties—they suppress the ROS formation during ischemia/reoxygenation and hypoxia; also they activate mitochondrial biogenesis and, as a result, maintain a normal level of intracellular ATP and Ca^2+^ homeostasis and promote cell survival in the penumbra zone [20,21]. Selenium can be accumulated in tissues and, in high doses, can show a cytotoxic effect on healthy tissues—these are the factors limiting its use in clinics. Selenium nanoparticles (SeNP) have a number of significant advantages compared with other Se-containing compounds. Selenium nanoparticles (SeNPs) are low toxic and have selective cytotoxicity: they lead cancer cells to their death and do not have an impact on normal cells [22].

In recent decades, the role of nanoparticles in neurological diseases has been actively studied, especially because of the fact that neurons are particularly vulnerable to the damage caused by oxidative stress due to high oxygen consumption, the presence of a large amount of polyunsaturated fatty acids, and the low expression of antioxidant enzymes [23,24]. Both “naked” selenium nanoparticles and nanoparticles doped with active compounds have been shown to be highly effective for the treatment of oncological diseases [22,25,26], neurodegenerative diseases [1,27,28,29], and ischemia [21,30].

The recommended dose of daily intake of taxifolin as a dietary supplement in the form of capsules or powder is 500–1000 mg/day for short-term courses. Higher-dose intake may result in side effects, such as headaches, stomachaches, and tingling sensations [31]. Similarly, excessive intake of selenium can lead to toxic effects, and its beneficial effects can be reversed [32,33]. In order to avoid the toxic effect of high doses of both selenium and taxifolin, and to increase their bioavailability, as well as targeted delivery, we developed a nanocomplex of selenium with taxifolin. A comparative analysis of the neuroprotective efficacy of the selenium nanocomplex with taxifolin in ischemia/reoxygenation was carried out in this study.

## 2. Materials and Methods

Experimental protocols were approved by the Bioethics Committee of the Institute of Cell Biophysics. Experiments were carried out according to Act708n (23 August 2010) of the Russian Federation National Ministry of Public Health, which states the rules of laboratory practice for the care and use of laboratory animals, and the Council Directive 2010/63 EU of the European Parliament on the protection of animals used for scientific purposes. 

### 2.1. Preparation and Characterization of Selenium Nanoparticles

**Figure 1:**
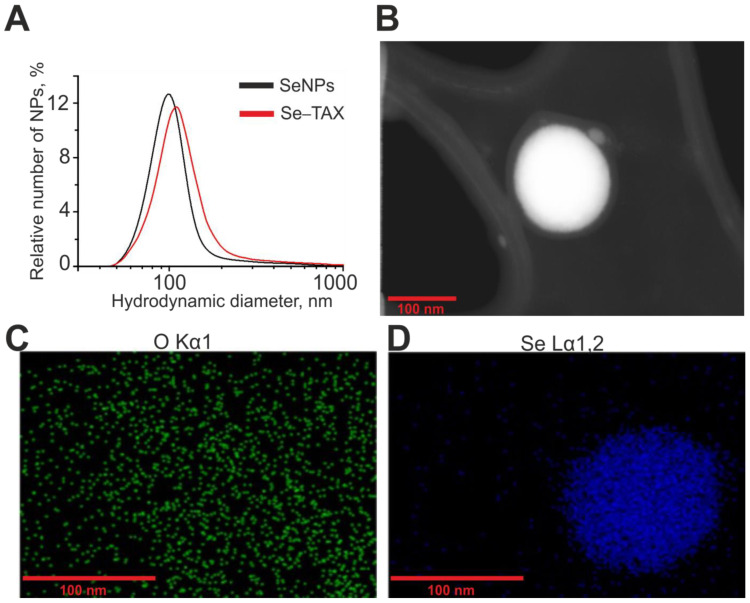
Shape, chemical composition, and size distribution of SeNPs and Se–TAX. (**A**) Size distribution of SeNPs (black) and Se–TAX. Data obtained using an analytical disk centrifuge and confirmed by DLS. (**B**) TEM image of an individual SeNP with a diameter of about 100 nm. (**C**) The distribution of oxygen atoms in panel (**B**). (**D**) The distribution of selenium atoms in panel (**B**).

Selenium nanoparticles were obtained by laser ablation in deionized water (18 MΩ cm). A massive selenium target was placed at the bottom of a cuvette under a layer of water a few millimeters thick. The target surface was exposed to a laser beam (λ = 1064 nm; T = 4–200 ns; f = 20 kHz; P = 20 W; Ep = 2 mJ). The laser beam was moved along the target surface using an LScanH galvanomechanical scanner (Ateko-TM, Moscow, Russia) along a given trajectory in the form of parallel straight lines inscribed in a rectangle. Depending on the characteristics of laser radiation and the speed and trajectory of the laser beam, it is possible to obtain colloidal solutions of selenium nanoparticles with specified geometric parameters. The size of nanoparticles was characterized using a DC24000 analytical centrifuge (CPS Instruments, Prairieville, LA, USA). The concentration of nanoparticles and the hydrodynamic radius were evaluated using the Zetasizer Ultra Red Label (Malvern, UK). It was found that the resulting preparation of selenium nanoparticles has a monomodal size distribution (Figure 1A). The average size of nanoparticles is about 100 nm, the half-width is in the range of 75–125 nm, and the zeta potential of nanoparticles is about −20 mV. The morphology of nanoparticles was studied by electron energy loss spectroscopy using a 200FE transmission electron microscope (Carl Zeiss, Oberkochen, Germany). According to electron microscopy data, nanoparticles have a spherical shape (Figure 1B). Figure 1C,D shows the distribution of oxygen and selenium atoms in the TEM image presented in the panel of Figure 1B. The microscope is equipped with an attachment for energy dispersive X-ray spectroscopy. The method of energy dispersive X-ray spectroscopy confirms that the obtained nanoparticles consist of selenium. 

Taxifolin (Cas#24198-97-8, Sigma-Aldrich, Burlington, NJ, USA) was dissolved in deionized water at a concentration of 1 g/L using a heated magnetic stirrer. During dissolution, heating was carried out at a rate of 10 °C/min to 60 °C. At this temperature, taxifolin dissolves in deionized water. A freshly prepared colloidal solution of SeNPs in deionized water with a nanoparticle concentration of 318 μg/mL was used to create the Se–TAX nanocomplex. Similarly, upon heating to 60 °C and stirring, taxifolin was added to the colloidal solution of nanoparticles, and the final concentration of taxifolin was 0.1 mg/mL. The heating was turned off, and a few drops of 0.1 M NaCl solution were added to the solution. After that, a container with a solution containing selenium nanoparticles and taxifolin was transferred “on ice”. The solubility of taxifolin in water depends on the pH. According to our data, the maximum solubility values were observed at a pH of about 4. Alkalinization and a decrease in temperature led to a significant decrease in solubility. As a result, taxifolin was deposited on the walls of the vessel and the surface of the nanoparticles. Selenium nanoparticles with taxifolin deposited on them were isolated from the solution by centrifugation. The supernatant was heated again, and the spectrum was taken. The amount of taxifolin associated with nanoparticles was determined from the change in the absorption spectrum of the solution before the addition of nanoparticles and after the deposition of nanoparticles. The procedure was carried out three times. The hydrodynamic radius of selenium nanoparticles after adding taxifolin increased to 110 nm and broadened (Figure 1A). A complex of physical methods was used to characterize the Se–TAX complex. The absorption spectrum of Se–TAX was measured on a Cintra 4040 (GBC Cintra, Melbourne, Australia) in quartz cuvettes with an optical path length of 10 mm at room temperature (~22 °C). The ζ-potential distribution of Se–TAX was registered with a dynamic light scattering method (DLS) on the Zetasizer Ultra Red Label (Malvern Panalytical, Malvern, UK). He–Ne laser with a wavelength of 633 nm and a scattering angle of 173° was used as a light source. Refractive index measurements were carried out on a multiwavelength refractometer: Abbemat MW (Anton Paar, Graz, Austria). In the experiments, 1 mL of the solution was poured into the cell of the device, and measurements were made at wavelengths of 435.8, 589.3, and 632.8 nm at a temperature of 25 °C. Se–TAX was studied on an FSM-2202 IR-Fourier spectrometer in the range of 600–1500 cm^–1^ (Fourier-transform infrared spectrometry). One beam of IR radiation passes through the cuvette compartment of this spectrometer, so it was necessary to sequentially obtain the comparison spectrum and the spectrum of the sample under study. The diameter of the IR beam in the constriction (in the middle of the cell compartment) was 11 mm. The comparison spectrum was the transmission spectrum of a clean germanium plate with dimensions of 19 × 49 mm. The plate was placed vertically in the beam constriction so that the beam passed through its middle. A Se–TAX solution with a volume of 450 μL was applied with a calibrated Biohit Proline AR16954 dispenser to the middle of the germanium plate in the form of a film with dimensions of ~15 × 19 mm. The films were dried at a temperature of 24 °C. The spectra were recorded with a resolution of 2 cm^–1^ and 50 averaging.

**Figure 2:**
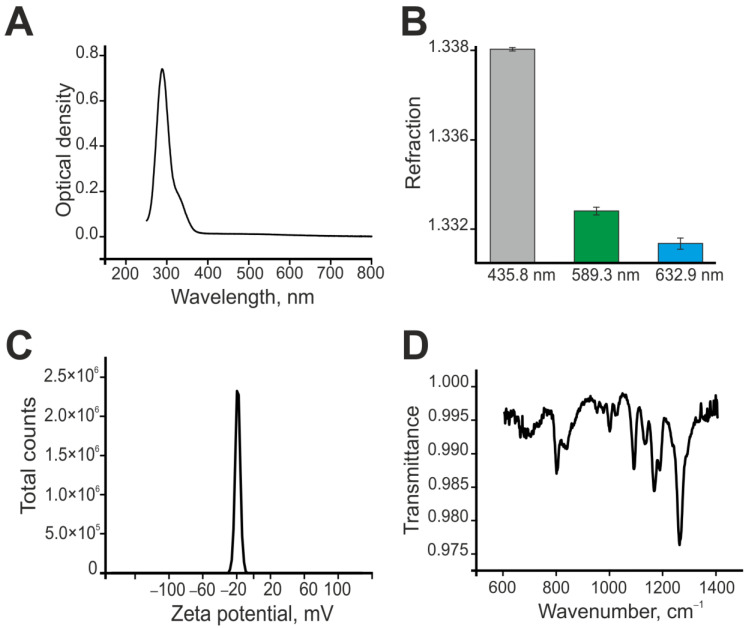
Shape, chemical composition, and size distribution of SeNPs and Se–TAX. (**A**) The absorption spectrum of the Se–TAX complex. (**B**) The refraction index of the Se–TAX complex. (**C**) The zeta potential of the Se–TAX complex. (**D**) The FTIR spectrum of the SeNP-TAX complex.

First of all, the absorption spectrum of the Se–TAX sample was obtained; it is represented in Figure 2A. The maximum of absorption is located on the 289 nm wavelength. The peak shows a slight extension on the 320–360 nm. Additionally, the refraction index of Se–TAX was registered; the data are represented in Figure 2B. The zeta potential increased to −20 mV in the Se–TAX complex (Figure 2C). Additionally, the Se–TAX complex was studied with the use of FTIR spectroscopy. The resulting FTIR spectrum is represented in Figure 2D. It shows the dependence of transmittance from the wavenumber. The presented spectrum of the Se–TAX complex contains the bands specific for the bond vibrations in the Se–TAX complex. Data on the microscopic examination of nanoparticles with adsorbed taxifolin molecules are not carried out, since the taxifolin “fur coat” has an extremely low contrast. 

### 2.2. Preparation of Mixed Neuroglial Cell Cultures

Cell cultures from the cerebral cortex were prepared as described in detail previously [34]. Briefly, 0- to 1-day-old mouse pups were euthanized and decapitated. The extracted tissue was washed with a Mg^2+^- and Ca^2+^-free Versene solution and minced with scissors. Then, the tissue fragments were digested with a 1% trypsin solution for 10 min at 37 °C and washed two times with a cold Neurobasal-A medium. Trypsinized tissue was gently triturated with a pipette, and the debris was then carefully removed with a pipette tip. The obtained cell suspension was seeded on polyethyleneimine-coated glass cover slips and grew for 10 days in vitro in the cell culture medium composed of the Neurobasal-A medium, supplement B-27 (2%), and 0.5 mM glutamine. 

The drugs were added into a culture medium under sterile conditions in the case of experiments with 24 h pre-incubation with TAX, SeNPs, and Se–TAX. Then, the cell cultures were washed after the preincubation with Hank’s balanced salt solution and used in experiments. 

### 2.3. Measurement of ROS Production

For simultaneous recordings of ROS production, cortical cell cultures were loaded with H2DCF-DA (10 µM, 20 min incubation; 37 °C). In addition, in order to evaluate the intensity of mitochondrial staining of the ROS indicators in the performed experiments, the double staining of cell cultures with H_2_DCF-DA/MitoTracker Red FM was performed. The final concentration of MitoTracker Red FM was 200 nM (20 min incubation; 37 °C). After incubation with the dyes, cells were washed three times before the experiment. To measure the ROS generation, the system based on the inverted motorized microscope Leica DMI6000B with a high-speed monochrome CCD-camera HAMAMATSU C9100 and a high-speed light filter replacing system Leica’s Ultra-Fast Filter Wheels with a replacing time 10–30 ms was used. For the excitation of DCFH2-DA, an L5 filter set (Leica, Germany) with the excitation filter BP480/40, dichroic mirror 505, and emission filter 527/30 was used. To evaluate H2DCF-DA/MitoTracker Red colocalization, an inverted confocal microscope (Leica TCS SP5, Wetzlar, Germany) was used. For the excitation of DCFH2-DA, an argon laser with line 488 nm was used. Emission was collected in the range of 510–590 nm. MitoTracker Red FM is a far-red-fluorescent dye; therefore, a 633 nm He–Ne laser for its excitation was used. Emission was collected in the range of 655–700 nm. In order to prevent nonspecific photo-oxidation of the ROS indicators, laser power was decreased to 3%–5%. The shape and speed of ROS production rates under oxygen–glucose deprivation (OGD) or application of H_2_O_2_ were determined. 

To register the total production of ROS in cells and to reveal the concentration effects of TAX, SeNPs, or Se–TAX, the brain cortex cells were grown in 24-well plates for 9 days. Next, various concentrations of the studied compounds were added to the culture medium for 24 h, after that, the cells were washed and loaded with H_2_DCF-DA (20 µM, 30 min incubation; 37 °C). The cells were then washed with HBSS, and baseline DCF fluorescence was recorded using an automated multiplate reader (Spark™ 10M multimode microplate reader; Tecan Trading AG, Switzerland, with the SparkControl 3.2 software). After detecting the base level of the fluorescence intensity, H_2_O_2_ at a concentration of 100 µM was added to initiate the ROS production. Recording was performed during 30–40 min. To avoid photo destruction of the probe and to avoid the photodynamic production of ROS, DCF fluorescence was recorded once every 5 min. The ImageJ, Origin 8.5, and Prism GraphPad software were used in order to analyze data, create graphs, and perform statistical tests. All values are given as mean ± SEM. All presented data were obtained from at least three cover slips and two to three different cell preparations.

### 2.4. Fluorescent Ca^2+^ Measurements

To detect the changes in [Ca^2+^]i, cell cultures were loaded with Fura-2 (4 µM; 40 min incubation; 37 °C). The cells were stained with the probe dissolved in Hank’s balanced salt solution (HBSS) composed of (mM): 156 NaCl, 3 KCl, 2 MgSO_4_, 1.25 KH_2_PO_4_, 2 CaCl_2_, 10 glucose, and 10 HEPES, pH 7.4. To measure [Ca^2+^]i, we used the system based on an inverted motorized microscope, Leica DMI6000B, with a high-speed monochrome CCD-camera HAMAMATSU C9100. For excitation and registration of Fura-2 fluorescence, we used an FU-2 filter set (Leica, Germany) with the excitation filters BP340/30 and BP387/15, beam splitter FT-410, and emission filter BP510/84. Illuminator Leica EL6000 with a high-pressure mercury lamp was used as a source of excitation light. To distinguish neurons and astrocytes, we used short-term applications of 35 mM KCl before the main experiments. This method is described in detail in our previous work [35]. Briefly, KCl induces depolarization of excitable cells, which contain a wide range of voltage-gated cation channels. KCl-induced depolarization promotes the opening of voltage-gated calcium channels in neurons (predominantly L-type channels). The conductivity and density of cation channels in astrocytes are insufficient to evoke high-amplitude Ca^2+^ response to KCl application. All the Ca^2+^ signals are presented as a 340/380 ratio of Fura-2 fluorescence.

### 2.5. Simulation of Ischemia-like Conditions

Ischemia-like conditions (oxygen–glucose deprivation, OGD) were obtained by omitting glucose (HBSS medium without glucose) and by displacement of dissolved oxygen with argon in the leak-proof system [34]. The level of oxygen in the medium was measured using a Clark electrode. Oxygen tensions reached values of 30–40 mm Hg or less within 20 min after the beginning of displacement. Ischemia-like conditions lasting for 40 min or 2 h were created by supplying the oxygen–glucose deprivation (OGD) medium into the chamber with cultured cortical cells. Constant argon feed into the experimental chamber was used to prevent the contact of the OGD medium with the atmospheric air. 

### 2.6. Assessment of Cell Viability and Apoptosis

Propidium iodide (1 µM) was used to evaluate the number of dead cells in the cell cultures before and after OGD. The cells were stained for 5 min with the probes diluted in HBSS and then rinsed with HBSS. Fluorescence of the probes was detected with an inverted fluorescent microscope, Zeiss Axio Observer Z1, using Filter Set 20. Cell death induced by OGD was assessed by propidium iodide staining (PI, 1 µM) before and after the exposures in the same microscopic field. Since PI stains both dead astrocytes and neurons, analysis of calcium signals upon 35 mM KCl application before OGD was used to identify the type of cells. Neurons were identified by the fast transient calcium signal upon KCl application, as described previously. Furthermore, we used the Ca^2+^ signals (presence or absence of a global increase in [Ca^2+^]i during OGD) as an additional indicator of cell viability [36]. 

Hoechst 33342 (2 µM) and propidium iodide (1 µM) were used to evaluate the number of dead cells in the cell cultures before and after 2 h OGD and 24 h reoxygenation. The cells were stained for 5 min with the probes diluted in HBSS and then rinsed with HBSS. Fluorescence of the probes was detected with an inverted fluorescent microscope, Zeiss Axio Observer Z1, using Filter Set 01 and Filter Set 20. Discrimination of early and late apoptotic cells was performed according to the previously described method [37]. Five different areas of each cell culture were analyzed. Each experimental group consisted of three cell cultures from different passages.

### 2.7. Extraction of RNA

A MagJET RNA Kit (Thermo Fisher Scientific, Waltham, MA, USA) was used for the extraction of total RNA. The RNA quality was estimated by electrophoresis in the presence of 1 μg/mL ethidium bromide (2% agarose gel in Tris/borate/EDTA buffer). The concentration of the extracted RNA was determined with a NanoDrop 1000c spectrophotometer. A RevertAid H Minus First Strand cDNA Synthesis Kit (Thermo Fisher Scientific, USA) was used for reverse transcription of total RNA.

### 2.8. Quantitative Real-Time Polymerase Chain Reaction (RT-qPCR) 

Each PCR was performed in a 25 μL mixture composed of 5 μL of qPCRmix-HS SYBR (Evrogen, Moscow, Russia), 1 μL (0.2 μM) of the primer solution, 17 μL of water (RNase-free), and 1 μL of cDNA. Dtlite Real-Time PCR System (DNA-technology, Moscow, Russia) was used for amplification. The amplification process consisted of the initial 5 min denaturation at 95 °C, 40 cycles of 30 s denaturation at 95 °C, 20 s annealing at 60–62 °C, and 20 s extension step at 72 °C. The final extension was performed for 10 min at 72 °C. All the sequences were designed with the FAST PCR 5.4 and NCBI Primer-BLAST software. The data were analyzed with the DTlite software (DNA-Technology, Moscow, Russia). The expression of the studied genes was normalized to gene-encoding glyceraldehyde 3-phosphate dehydrogenase (GAPDH). Data were analyzed using Livak’s method.

### 2.9. Statistical Analysis

All presented data were obtained from at least three cell cultures from two to three different passages. All values are given as mean ± standard error (SEM) or as individual cellular signals in experiments. Statistical analyses were performed by paired *t*-test. Differences are significant at * *p* < 0.05, ** *p* < 0.01, and *** *p* < 0.001. n/s—data not significant (*p* > 0.05). MS Excel, ImageJ, Origin 2016 (OriginLab, Northampton, MA, USA), and Prism GraphPad 7 (GraphPad Software, RRID: SCR_002798) software were used for data and statistical analysis.

## 3. Results

### 3.1. Dose-Dependent Effect of Taxifolin (TAX), Selenium Nanoparticles (SeNPs), and Selenium–Taxifolin Nanocomplex (Se–TAX) on H_2_O_2_-Induced ROS Production in Cerebral Cortex Cells

**Figure 3:**
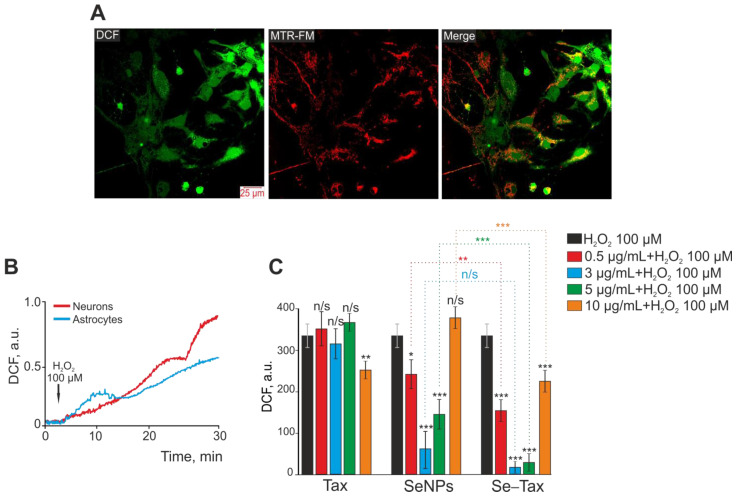
Dose-dependent effect of 24 h preincubation of cerebral cortex cells with taxifolin (TAX), selenium nanoparticles (SeNPs), and selenium–taxifolin nanocomplex (Se–TAX) on ROS production induced with the addition of 100 μM H_2_O_2_. (**A**) Simultaneous staining of cerebral cortex cells with a DCF probe (to measure ROS production) and a mitochondrial probe (MitoTracker Red FM, MTR-FM) and their combination (Merge). (**B**) ROS production in neurons (red curve) and astrocytes (blue curve) of the cerebral cortex in culture with the addition of 100 μM H_2_O_2_. The curves of ROS production averaged over several dozens of cells, recorded by increasing the fluorescence of the DCF probe, are presented. (**C**) Effect of 24 h cell incubation with various concentrations of TAX, SeNPs, and Se–TAX on H_2_O_2_-induced ROS production. Data obtained with an automated multiplate reader (Spark™ 10M multimode microplate reader) are presented. Data are shown as the mean of fluorescence intensity, arb.units ± S.E.M. Statistical analysis of experimental groups versus H_2_O_2_ (black bars) was performed with paired *t*-test. Significance between group means: *** *p* < 0.001, ** *p* < 0.01 and * *p* < 0.05. n/s—insignificant differences.

An increase in DCF fluorescence (H2DCFDA, 2′,7′-dichlorodihydrofluorescein diacetate) represents total ROS production by the cerebral cortex cells since this probe was localized both in the cytosol (Figure 3A, DCF) and in mitochondria (Figure 3A, MTR-FM; Figure 3A, Merge). Exogenous H_2_O_2_ mimics oxidative stress, which promotes pathological processes; thus, H_2_O_2_-induced cell injury is a good in vitro model of neurotoxicity induced by oxidative stress [38,39]. Exogenous H_2_O_2_ at a concentration of 100 µM induced ROS production by neurons and astrocytes of the cerebral cortex in a cell culture (Figure 3A). In both neurons (Figure 3B, red curve) and astrocytes (Figure 3B, blue curve), the increase of ROS production occurred biphasically within 30 min of DCF fluorescence recording. However, the increase in ROS production was more pronounced in the neurons of the cerebral cortex. Previously, the effectiveness of taxifolin for suppressing ROS production in brain cortex cells in OGD has been shown [10]. Moreover, selenium nanoparticles (SeNPs) showed high cytoprotective efficacy in OGD and other damaging changes on brain cells [40].

Preincubation of cells with TAX at concentrations of 0.5–5 µg/mL did not affect H_2_O_2_-induced ROS production, and only the concentration of 10 µg/mL TAX led to a significant suppression of ROS (Figure 3C, TAX). However, 24 h preincubation with SeNPs led to the suppression of ROS already at a concentration of 0.5–5 µg/mL, whereas 10 µg/mL SeNPs did not affect H_2_O_2_-induced ROS growth (Figure 3C, SeNPs). The selenium–taxifolin nanocomplex was more effective in the suppression of ROS production compared with the same concentrations of SeNPs. It is especially interesting that a high Se–TAX concentration (10 µg/mL) also suppressed ROS growth (Figure 3C, Se–TAX) in contrast with “naked” SeNPs.

Therefore preincubation of cerebral cortex cells with selenium nanoparticles in the concentration range of 0.5–5 μg/mL led to the suppression of H_2_O_2_-induced ROS production in cerebral cortex cells, with the greatest effect of ROS suppression at a concentration of 3 μg/mL SeNPs. The selenium–taxifolin nanocomplex significantly suppressed ROS production in the concentration range of 0.5–10 μg/mL with the addition of H_2_O_2_ compared with “naked” SeNPs. The selenium–taxifolin nanocomplex is deprived of the effect of high doses, which is characteristic for “naked” SeNPs. Thus, at a concentration of 10 μg/mL, Se–TAX suppressed ROS production and did not cause any toxic effect on brain cells. Taxifolin in a similar concentration range had no effect on H_2_O_2_-induced ROS production. A little effect of ROS suppression in cells was observed only after incubation with 10 μg/mL taxifolin.

### 3.2. Effects on the Calcium Dynamics in Astrocytes of the Cerebral Cortex

**Figure 4:**
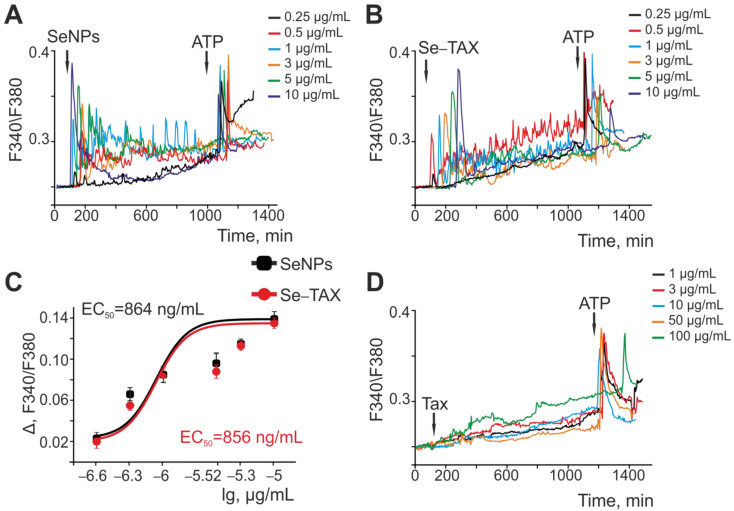
Activation of the Ca^2+^ signaling system of cortical astrocytes by different concentrations of SeNPs (**A**) and Se–TAX (**B**). (**A**,**B**) Application of various concentrations of SeNPs (**A**) and Se–TAX (**B**) induces the generation of Ca^2+^ signals in the cortical astrocytes. Ca^2+^ signals in astrocytes averaged over several dozens of cells in one experiment are presented. (**C**) Dependence of the amplitude of Ca^2+^ responses of astrocytes on the growth concentration of SeNPs (black squares) and Se–TAX (red circles) and its approximation by a sigmoid function. (**D**) Application of various concentrations of taxifolin (TAX) does not cause the generation of Ca^2+^ signals by astrocytes of the cerebral cortex.

It has been shown previously that selenium nanoparticles activate the Ca^2+^-signaling system of astrocytes from the cerebral cortex, and the effects of SeNPs on neurons are mediated by neuron–glial interactions [18]. “Naked” SeNPs (Figure 4A) and the Se–TAX nanocomplex (Figure 4B) generated Ca^2+^ signals in astrocytes, and the amplitude of these signals increased with increasing concentration of nanoparticles. The amplitude of the Ca^2+^ responses of astrocytes with SeNPs (Figure 4C, black squares) and Se–TAX (Figure 4C, red circles) depended on their concentration and showed that both types of nanoparticles induced almost similar Ca^2+^ signals. EC50 for SeNPs was 864 ng/mL, and for the Se–TAX nanocomplex, it was 856 ng/mL (Figure 4C). Taxifolin application at concentrations of 1–50 μg/mL did not cause the generation of high-amplitude Ca^2+^ signals in astrocytes; only a moderate increase in baseline [Ca^2+^]i was observed. The addition of 100 μg/mL taxifolin caused a significantly more pronounced increase in [Ca^2+^]i, compared with lower concentrations (Figure 4D).

Therefore, taxifolin at the studied concentrations did not cause the generation of high-amplitude Ca^2+^ signals in the astrocytes of the cerebral cortex, but at high doses (starting from 100 μg/mL) it contributed to an increase in the base concentration of [Ca^2+^]i. The application of “naked” SeNPs or the Se–TAX nanocomplex caused dose-dependent generation of Ca^2+^ signals in astrocytes, including an increase in the base concentration of [Ca^2+^]i, Ca^2+^ oscillations, and other modes of behavior of the Ca^2+^ signaling system. Note that SeNPs and Se–TAX showed similar EC50 values. This indicates the effect of nanoselenium on the Ca^2+^ signaling system of astrocytes, but not the taxifolin adsorbed on the nanoparticles.

### 3.3. SeNPs and Se–TAX Suppress ROS Production and Generate a Global Increase in [Ca^2+^]i during Ischemia

**Figure 5:**
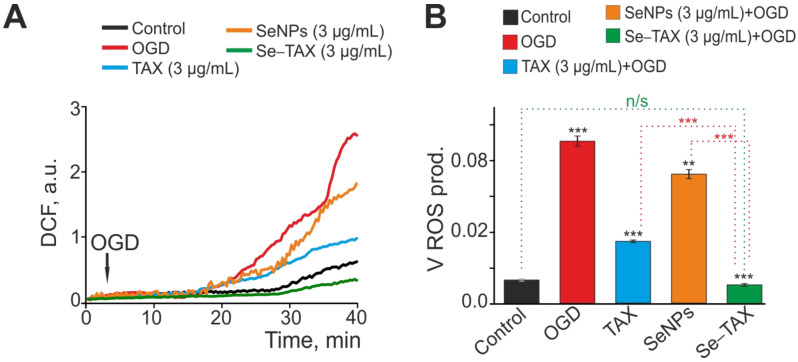
Effects of taxifolin (TAX), selenium nanoparticles (SeNPs), and selenium–taxifolin nanocomplex (Se–TAX) on OGD-induced ROS production. (**A**) Total ROS production during OGD (40 min) depending on a 24 h incubation of the cell cultures with 3 μg/mL TAX, 3 μg/mL SeNPs, or 3 μg/mL Se–TAX. Average curves plotted according to data from several dozen cells are represented. (**B**) Effect of the TAX, SeNPs, or Se–TAX on the ROS production rate. The average ROS production rates within the interval from the point of OGD onset to the end point of the experiment are represented. n/s—data not significant (*p* > 0.05), ** *p* < 0.01, and *** *p* < 0.001, compared with control.

As presented above, “naked” SeNPs and the Se–TAX nanocomplex showed the greatest efficiency in suppressing H_2_O_2_-induced ROS production at a concentration of 3 μg/mL. Additionally, EC50 for activating the Ca^2+^ signaling system of astrocytes was about 1 μg/mL. This result implies the use of a higher concentration of nanoparticles in experiments for more efficient activation of Ca^2+^ responses. Therefore, we used SeNPs and Se–TAX at a concentration of 3 μg/mL in further experiments. During OGD, we revealed two phases of an increase in ROS production. The first phase occurs 15–20 min after the OGD induction, and the second can be observed as the increase in ROS production after 30–35 min of DCF fluorescence registration (Figure 5A, OGD). Preincubation of cells for 24 h with TAX resulted in the suppression of the second phase of the OGD-induced ROS burst (Figure 5A, TAX) and the decrease in ROS production rate by more than two times (Figure 5B). Preincubation with “naked” SeNPs also suppressed the second phase of OGD-induced ROS growth (Figure 5A, SeNPs); however, the decrease in ROS production rate was less pronounced compared with TAX (Figure 5B). The Se–TAX nanocomplex (Figure 5A,B, Se–TAX) proved to be the most effective in suppressing OGD-induced ROS production, attenuating ROS generation up to control levels.

**Figure 6:**
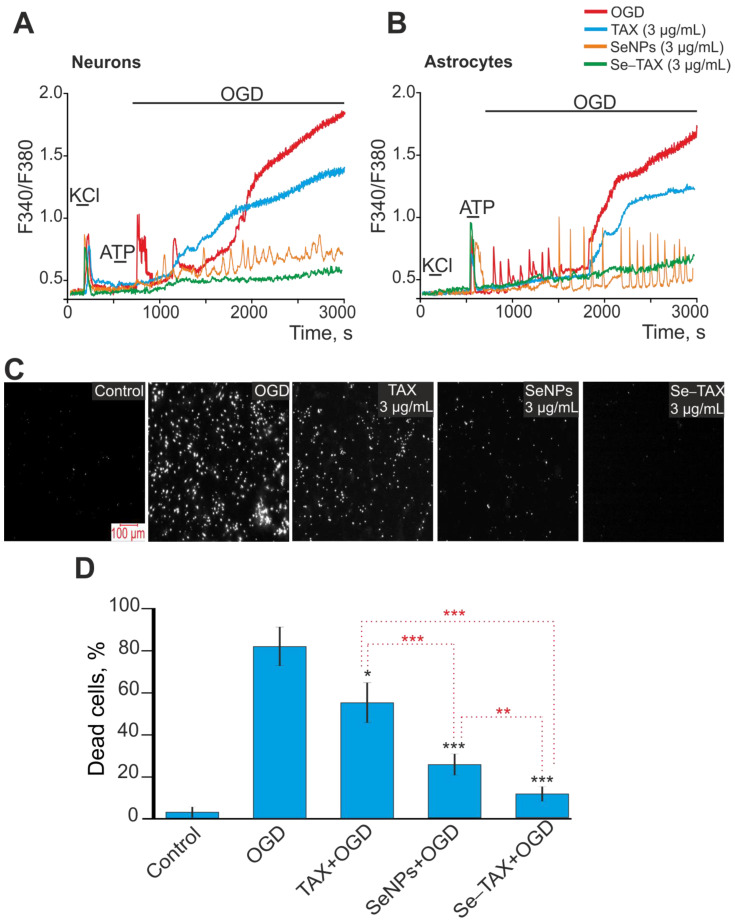
Effect of preincubation of cerebral cortex cells with TAX, SeNPs, or Se–TAX on Ca^2+^ signal generation and survival after OGD. (**A**,**B**) Ca^2+^ signals in neurons (**A**) and astrocytes (**B**) during OGD (40 min) and OGD after 24 h preincubation with 3 μg/mL taxifolin, 3 µg/mL SeNPs, or 3 µg/mL Se–TAX. Short-term applications of 35 mM of KCl and 10 µm of ATP were used to distinguish neurons and astrocytes, respectively. Ca^2+^ signals from neurons and astrocytes averaged over several dozens of cells are presented. The experiments were performed in three replicates on three separate cell cultures. (**C**) Images of cortical cell culture loaded with propidium iodide (PI). The white dots represent the PI-stained nuclei of necrotic cells. (**D**) Effect of 24 h incubation with TAX, SeNPs, or Se–TAX on the cell viability after 40 min OGD. Black asterisks indicate the differences between the experimental groups compared with the OGD group. Differences between experimental groups are marked with red asterisks. *** *p* < 0.001, ** *p* < 0.01, and * *p* < 0.05.

In OGD, there was a biphasic increase of Ca^2+^ ions in the cytosol of neurons (Figure 6A) and astrocytes (Figure 6B). This led to cell death of 80% or more of the cells due to necrosis (Figure 6C,D, OGD), whereas for the control, cell death was about 10%, registered with the fluorescence of propidium iodide (Figure 6C,D, control). Preincubation of cortical cells with 3 μg/mL TAX inhibited the first reversible phase of the OGD-induced increase of [Ca^2+^]i in neurons (Figure 6A, TAX) and astrocytes (Figure 6B, TAX). However, it did not suppress the second phase of the global increase in [Ca^2+^]i. OGD-induced cell death was reduced up to 60% after incubation with TAX (Figure 6C,D). Incubation of cells with “naked” SeNPs suppressed the first and second phases of the OGD-induced increase in [Ca^2+^]i in neurons (Figure 6A, SeNPs) and astrocytes (Figure 6B, SeNPs). However, signs of ischemic hyperexcitation in the form of stable Ca^2+^ oscillations persisted in both cell types. Cell death after OGD was reduced up to 20% under the treatment by SeNPs (Figure 6C,D). The Se–TAX nanocomplex not only inhibited the first and second phases of the OGD-induced increase in [Ca^2+^]i in neurons (Figure 6A, Se–TAX) and astrocytes (Figure 6B, Se–TAX), but also suppressed the hyperexcitation. Cell death after incubation with Se–TAX was 8%–15% after OGD (Figure 6C,D), which was close to control values.

Therefore under ischemia-like conditions (OGD), the selenium–taxifolin nanocomplex showed the highest efficiency in suppressing ROS production, inhibiting the global increase in [Ca^2+^]i, hyperexcitation, and necrotic cell death. The preincubation with this complex reduced cell death to control values. Meanwhile, taxifolin at a concentration of 3 μg/mL was able to suppress the second phase of ROS production (as well as “naked” SeNPs) during ischemia and had almost no effect on the Ca^2+^ dynamics. As a result, it was significantly less effective in suppressing necrosis.

### 3.4. The Cytoprotective Effect of SeNPs and Se–TAX in Cerebral Cortex Cells during Ischemia/Reoxygenation Associated with Changes in Redox Status and Suppression of Apoptosis and Inflammation Pathways

**Figure 7:**
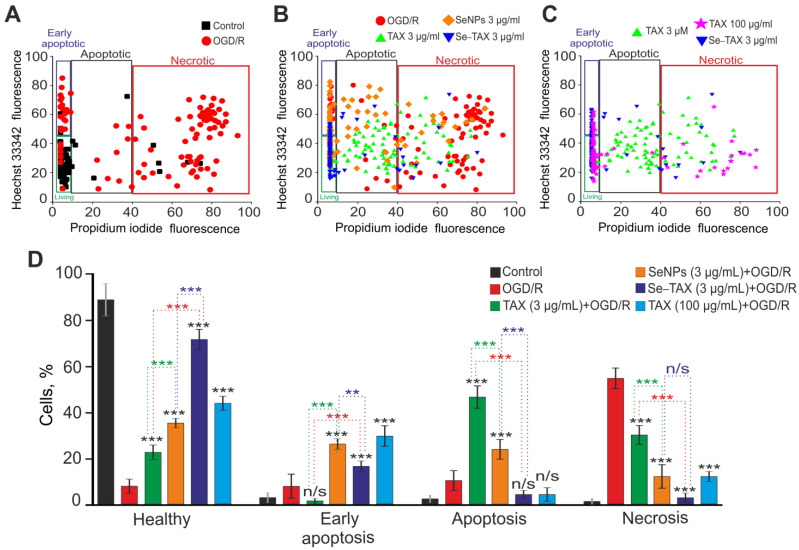
The effects of 24 h cell incubation with TAX (3 or 100 μg/mL), SeNPs (3 µg/mL), or Se–TAX (3 µg/mL) on the survival of cerebral cortical cells after 2 h OGD and 24 h reoxygenation (OGD/R). (**A**–**C**) Cytograms demonstrating the viability of cortical primary cultured cells in control (without OGD/R) and after OGD/R (**A**), after OGD/R with TAX (3 μg/mL), SeNPs (3 µg/mL), or Se–TAX (3 µg/mL) (**B**) and after OGD/R with TAX (3 or 100 μg/mL) and Se–TAX (3 µg/mL) (**C**). X-axis—the intensity of PI fluorescence; Y-axis—the intensity of Hoechst 33342 fluorescence. Cells were stained with the probes 24 h after the OGD/R. (**D**) Effect of 3 or 100 μg/mL TAX, 3 µg/mL SeNPs, or Se–TAX on the induction of necrosis and apoptosis 24 h after OGD/R. N cell cultures = 5; n cover slips with cells for each sample = 5. For panel (**D**), results are presented as mean ± SEM. Black asterisks indicate the differences between the experimental groups comparable to the OGD group. Differences between experimental groups are marked with asterisks of different colors. n/s—data not significant (*p* > 0.05), *** *p* < 0.001 and ** *p* < 0.01. Cell images are presented in Supplementary Figure S1.

OGD followed by 24 h reoxygenation (OGD/R) caused huge death of cerebral cortex cells due to the induction of early (14%) and late (16%) stages of apoptosis (Figure 7A,D). However, the necrosis (56%) was predominantly observed; it was detected with PI fluorescence (Supplementary Figure S1). In the control, without treatment, only single cells died—no more than 10% of the total number of cells in the field of view (Figure 7A,D, Control). After preincubation of cells with 3 μg/mL TAX followed by OGD/R, 22% of cells survived, 3% and 46% were in the early and late stages of apoptosis, and necrosis was shown in 29% of cells (Figure 7B,D; Supplementary Figure S1). Incubation with SeNPs promoted cell survival (33%), 27% and 23% of cells were in the early and late stages of apoptosis, and necrosis was monitored in 17% of cells (Figure 7B,D; Supplementary Figure S1). The incubation with Se–TAX resulted in the survival of 68% of cells after OGD/R and the inhibition of the early and late stages of apoptosis (18% and 8% of cells, respectively), and necrosis was recorded in 6% of cells (Figure 7B,D; Supplementary Figure S1). Interestingly, the use of high concentrations of TAX (100 μg/mL) resulted in cortical cell survival comparable to the use of 3 µg/mL SeNPs. After OGD/R with 100 μg/mL SeNPs, 46% of cells survived, the early and late stages of apoptosis were observed in 29% and 8% of cells, and necrosis was seen in 17% of cells (Figure 7C,D; Supplementary Figure S1). Preincubation of cells with 3 μg/mL TAX, SeNPs, or Se–TAX without exposure to OGD/R did not induce significant cell death (Supplementary Figure S2).

**Figure 8:**
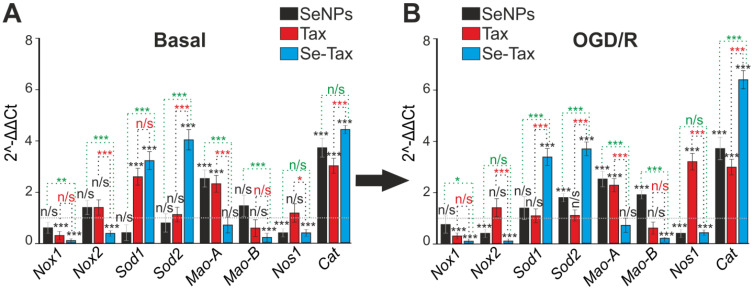
The effect of 24 h incubation of mice cortical cells with 3 μg/mL taxifolin (TAX), 3 μg/mL SeNPs, or 3 μg/mL Se–TAX on the basal (**A**) and OGD/R-affected (**B**) expression of genes regulating cellular antioxidant status. Gene expression in intact cells are marked with a dashed line for panel (**A**). Gene expression in OGD/R cells without treatment with test compounds is marked with a dashed line for panel (**B**). Statistical significance was assessed using *t*-test. Comparison of experimental groups with control: n/s—data not significant (*p* > 0.05), * *p* < 0.05, ** *p* < 0.01, and *** *p* <0.001. Comparison of experimental groups with each other is indicated in red or green. The number of samples is 3.

Survival of brain cells under conditions of ischemia/reoxygenation depends on the redox status of the cells. Using PCR analysis, it was found that TAX reduced the basic expression of Nox1 and increased the level of Sod1, Mao-A, and Cat (Figure 8A, TAX), indicating an increase in the antioxidant status of cells. SeNPs are less effective in regulating the antioxidant status of cells. Preincubation with 3 μg/mL SeNPs resulted in an increase in the baseline expression of Mao-A and Cat with a decrease in the level of Nos1 (Figure 8A, SeNPs). Incubation of cells with the Se–TAX nanocomplex resulted in a striking change of the basic expression of the genes that regulate the redox status. Two hours after incubation of cells with Se–TAX, the expression of most of the genes encoding ROS generating the proteins Nox1, Moa-A, Moa-B, and Nos1 was suppressed. This occurred simultaneously with an increased expression of genes encoding the ROS-consuming proteins Sod1, Sod2, and Cat (Figure 8A, Se–TAX).

Twenty-four hours after OGD/R, the expression profile of genes encoding redox status-associated proteins was changed. The incubation of cells with taxifolin led to positive changes due to the suppression of Nox1 expression and an increase in Cat. However, a significant increase in MAO-A and Nos1 expression may have a negative effect (Figure 8B, TAX). After the incubation of cells with SeNPs and OGD/R, the expression of Nox2 and Nos1 was suppressed, and the level of Sod2 and Cat increased. We interpret this as a beneficial effect, whereas an increase in Mao-A and Mao-B is a negative phenomenon and contributes to oxidative stress (Figure 8B, SeNPs). The Se–TAX nanocomplex and subsequent OGD/R resulted in the maximum (compared with TAX and SeNPs) suppression of all key pro-oxidant genes’ expression, Nox1, Nox2, Mao-B, and Nos1, and an increase in the levels of genes encoding ROS-neutralizing proteins, Sod1, Sod2, and Cat (Figure 8B, Se–TAX).

**Figure 9:**
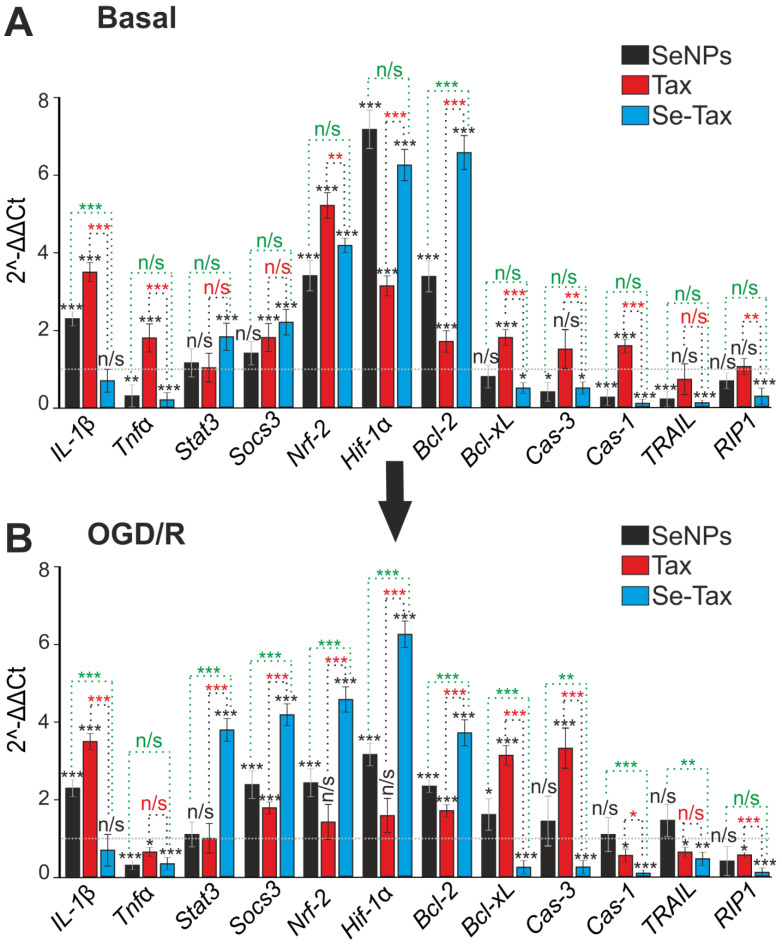
The effect of 24 h incubation of mice cortical cells with 3 μg/mL taxifolin (TAX), 3 μg/mL SeNPs, or 3 μg/mL Se–TAX on the basal (**A**) and OGD/R-affected (**B**) expression of genes that regulate cell death and inflammation. Gene expression in control cells is marked with a dashed line for panel (**A**). Gene expression in OGD/R cells without treatment with test compounds are marked with a dashed line for panel (**B**). Statistical significance was assessed using *t*-test. Comparison of experimental groups with control: n/s—data not significant (*p* > 0.05), * *p* < 0.05, ** *p* < 0.01, and *** *p* <0.001. Comparison of experimental groups with each other is indicated in red or green. The number of RNA samples is 3.

Redox status could regulate the induction of necrosis, apoptosis, and inflammation. Taxifolin after 24 h of incubation led to an increase in the level of basic expression of the IL-1β, Tnfα, Bcl-xL and Cas-1 genes. This can be considered a negative effect, but on the other hand, an increase in the expression of Socs3, Nrf-2, Hif-1α, and Bcl-2 can protect cells (Figure 9A, TAX). Incubation with SeNPs led to the suppression of the basic expression of proinflammatory and proapoptotic genes—Tnfα, Cas-3, Cas-1, and TRAIL. It also led to the simultaneous increase in the expression of protective genes—Nrf-2, Hif-1α, and Bcl-2 (Figure 9A—SeNPs). Moreover, the effect of SeNPs on gene expression is more pronounced than with TAX. The incubation of cells with the Se–TAX nanocomplex increased the expression of all protective genes, Stat3, Socs3, Nrf-2, Hif-1α, and Bcl-2, while suppressing almost all proinflammatory and proapoptotic genes, Tnfα, Bcl-xL, Cas-3, Cas-1, TRAIL, and RIP1 (Figure 9A, Se–TAX), significantly and more pronouncedly compared with the other two agents.

The incubation of cells with TAX and subsequent OGD/R affects the expression of only a few genes responsible for cell survival—the expression of the Tnfα, Cas-1, TRAIL, and RIP1 genes decreased. These genes are responsible for the transition of cells to the late stages of apoptosis or activation of necrosis. However, the expression of the antiapoptotic genes Socs3 and Bcl-2 increased (Figure 9B, TAX). At the same time, the expression of the proinflammatory IL-1β and proapoptotic Bcl-xL and Cas-3 genes also increased (Figure 9B, TAX). The incubation of cells with SeNPs had almost no effect on the Cas-1, TRAIL, and RIP1 genes, although the expression of proinflammatory IL-1β and proapoptotic Bcl-xL increased. SeNPs suppressed the OGD/R-induced expression of the proinflammatory Tnfα and increased the expression of the antiapoptotic genes Socs3, Nrf-2, Hif-1α, and Bcl-2 (Figure 9B, SeNPs). The cytoprotective effect of the Se–TAX nanocomplex was demonstrated under OGD/R, since the expression of all genes responsible for cell death decreased, and the level of expression of all protective genes increased several times (Figure 9B, Se–TAX).

Therefore the preliminary incubation of brain cortex cells with the selenium–taxifolin nanocomplex for 24 h led to a significant increase in the baseline and OGD/R-induced expression of genes encoding proteins responsible for ROS utilization, suppression of apoptosis, necrosis, and inflammation. This effect occurred with the background of a powerful suppression of pro-oxidant and proapoptotic proteins. The use of free taxifolin or selenium in the form of “naked” nanoparticles affected a significantly smaller number of genes, with taxifolin mainly affecting the redox status-related ones, and nanoselenium the apoptotic pathway. In addition, the single use of taxifolin or selenium nanoparticles activated some genes involved in the cell death induction. This probably determines a fairly large percentage of cells at various stages of apoptosis (Figure 7).

## 4. Discussion

Despite the high antioxidant efficiency of taxifolin, the problem of its poor solubility and its inefficient delivery across the blood–brain barrier to neurons remains unresolved. The solubility of taxifolin can be improved by its use in combination with nanoparticles. It is known that the solubility of bioactive molecules is determined with their shape and size, meaning that small particles have both a large surface area and dissolution rates [41,42]. However, too small or too large nanoparticles are toxic or are difficult to penetrate inside the tissues [40]. Usually, nanoparticles with a spherical shape and a size of about 100 nm show no aggregation. This fact makes them much suitable for drug delivery in order to target organs, especially the brain. So far, the brain has remained the ultimate challenge in drug delivery. It has been found that nanoparticulated taxifolin treatment prior to ischemic insult provides strong protection to enzymatic antioxidant systems, whereas free taxifolin shows weak improvement [43]. It was also found that 84 nm spherical nanoparticles containing 45% taxifolin added to MCF-7 cells at a concentration of 10 µM exhibit anticancer efficacy comparable to the addition of 100 µM of pure taxifolin. In addition, taxifolin in nanoparticles is excreted within 12 h by 67.2% in in vitro and in vivo models, whereas taxifolin without nanoparticles is rapidly metabolized and converted into other products that no longer have such antioxidant properties [44]. Similarly, in this study, the Se–TAX nanocomplex with a nanoparticle size of 100 nm demonstrated a cytoprotective effect in the concentration range of 0.5–10 µg/mL. In addition, taxifolin in the form of nanoparticles reduced the levels of inflammatory cytokines and prevented the decrease in the levels of anti-inflammatory IL-10. This effect is not observed for free taxifolin [45]. Increased anti-inflammatory efficacy and estrogenic activity of taxifolin-doped nanoparticles have been shown in a model of ovariectomized rats: nanoparticles were effective at 10 mg/kg and free taxifolin at 50 mg/kg [46]. As for the blood–brain barrier, nanoparticles easily penetrate it. At the same time, it is known that after a stroke, the blood–brain barrier is damaged. This contributes to its permeability to inflammatory factors, activation of ROS overproduction, neuronal damage, and cognitive dysfunctions. The use of taxifolin (25 µM/kg) improves the structure of the blood–brain barrier and ameliorates its dysfunction through activating the canonical Wnt/β-catenin signaling pathway in the rat cerebral ischemia/reperfusion model [47,48]. In a mice model of stroke, it was found that SeNPs enter brain cells via transferrin receptor-mediated endocytosis, inhibit the inflammatory response, and increase the survival of hippocampal neurons [49]. It was also found that SeNPs 100 nm in size, which were used to create the Se–TAX nanocomplex, are characterized by the highest efficiency in going inside the astrocytes of the cerebral cortex, simultaneously activating two endocytosis pathways [40]. Thus, doping of nanoparticles with taxifolin would contribute to a more effective restoration of the blood–brain barrier.

The suppression of ROS by both taxifolin and SeNPs is well known. SeNP, depending on the dose, possess both pro-oxidant and antioxidant properties. SeNPs at a concentration of 12 μM have been shown to promote the antioxidant capacity of cells, whereas a treatment of 24 μM SeNPs damaged the antioxidant capacity of cells [50]. The antioxidant action of SeNPs is realized through selenoproteins and a number of enzymes—glutathione peroxidase, thioredoxin reductase, and iodothyronine deiodinases. SeNPs scavenge wide range of ROS: superoxide anion, 1,1-diphenyl-2-picrylhydrazyl, singlet oxygen, and carbon-centered free radicals [51,52,53], meaning that SeNPs could suppress overall oxidative stress affecting mitochondrial and cytosolic sources of ROS.

Taxifolin consumption as a dietary supplement reduces the risk of a stroke [54]. In the present work, we showed that taxifolin, SeNPs, and Se–TAX were able to suppress the rate of ROS production during OGD or H_2_O_2_ treatment. Since the cells of the cerebral cortex were incubated with the studied compounds for 24 h before the experiments, the effects were determined by the change in the redox status of the cells. Taxifolin itself and in the form of nanoparticles protects cardiomyocytes from ischemia/reoxygenation by maintaining the mitochondrial membrane potential and ATP synthesis [55]. The PLGA–quercetin nanocomplex at a concentration of 5 μM suppresses mitochondrial O_2_- and H_2_O_2_ production by 22% and 83% more effectively than free taxifolin [56]. The antioxidant effect of taxifolin is observed in an increase in the expression of Cu/ZnSOD and MnSOD and reduced damage resulting from transient cerebral ischemic in hippocampal CA1 pyramidal neurons of gerbils [57]. In our experiments, 3 μM TAX increased the baseline expression of SOD1; however, the Se–TAX nanocomplex activated SOD1 more efficiently at a similar concentration, and also induced SOD2. Both naked SeNPs and Se–TAX increased baseline or OGD/R-induced SOD and catalase expression, whereas free taxifolin increased only catalase levels. Taxifolin has been shown to suppress mitochondrial ROS production in brain cells by a decrease in the expression and activity of monoamine oxidases [58]. However, the concentration of free taxifolin (3 μM) used in our study was not sufficient, and taxifolin did not affect the baseline expression, or even increased OGD/R induced the expression of monoamine oxidases (Mao-A and Mao-B). Naked SeNPs also did not affect the expression of these mitochondrial proteins. However, the Se–TAX nanocomplex effectively decreased the expression of monoamine oxidase B. Summarily, Se–TAX more effectively affected the expression of various genes in the cells of the cerebral cortex towards an increase in the antioxidant status, suppressing the level of pro-oxidant proteins, especially NOX and NOS. Increased expression and activity of NOS excessive release of nitric oxide (NO) has been implicated in neurodegeneration in ischemic stroke [57]. Maximum protection against ischemia-reperfusion-induced NOS expression in the hippocampus was observed in rats treated with nanoencapsulated taxifolin after operation or prior to ischemic insults [43].

The processes of ROS production, mitochondrial damage, and cell death are closely connected to the dynamics of [Ca^2+^]i. It has been shown that short-term or chronic consumption of taxifolin enhances the antioxidant status of hippocampal cells through increased expression of antioxidant enzymes Sod1 and catalase and suppression of the increase in Ca^2+^ ions in the cytosol [59,60]. It is known that an increase in the baseline levels of [Ca^2+^]i can lead to cell death when such an increase is irreversible [34,61] or causes disturbances in intracellular signaling [62]. Previously, we showed that high concentrations of TAX (100 μM) after 24 h exposure are able to suppress the global increase in [Ca^2+^]i in hippocampal cells during OGD [10]. In the present study, it is confirmed that low doses of TAX (3 μM) do not suppress the global increase in [Ca^2+^]i during OGD in cortical cells. However, they are able to inhibit reversible Ca^2+^ signals, which is a sign of neuronal hyperexcitation. It is known that ROS scavengers, and especially taxifolin, enhance the GABAergic component of neurotransmission during hypoxia and anoxia, as well as upon the addition of exogenous H_2_O_2_ [63]. This fact may explain the effect of suppressing the hyperexcitation in brain cortex cells using 3 μM free taxifolin, when the inhibitory component of neurotransmission improved. In addition, TAX does not penetrate the blood–brain barrier well, and achieving even low doses in the area of brain damage is problematic in a short time. SeNPs are able to easily penetrate the blood–brain barrier and have powerful antioxidant properties that can improve the protection of brain cells from ischemic damage [21,64]. Nevertheless, “naked” SeNPs do not suppress neuronal hyperexcitation, but completely inhibit the global increase in [Ca^2+^]i. However, the nanocomplex of selenium with taxifolin (Se–TAX) exhibits the beneficial features of both components, suppressing both the total increase in [Ca^2+^]i and ischemic hyperexcitation of the neuronal network.

A cascade of damaging intracellular events triggered with ischemia/reoxygenation in brain cells ultimately leads to the induction of necrosis and apoptosis. Previously, we showed that prolonged incubation of cerebral cortex cells with SeNPs leads to the inhibition of the OGD-induced global increase of [Ca^2+^]i and necrotic death through the activation of reactive astrogliosis and the expression of genes encoding cytoprotective proteins [18]. In this study, all compounds applied at the same concentrations showed a high cytoprotective effect on OGD/R, but suppressed necrosis with different efficiency, while the Se–TAX nanocomplex was the most effective in inhibiting both necrosis and apoptosis. Recently, it was shown that treatment with taxifolin activates the mRNA expression of NRF2 with the translocation of NRF2 to the nucleus and protects RPE cells against oxidative-stress-induced apoptosis [65]. After OGD/R, SeNPs and Se–TAX showed significant Nrf-2 activation, in contrast to free TAX. The protective role of STAT3 (which is upregulated in the particular Se–TAX experiments) is closely associated with Bcl-xL, Bcl-2, and survivin. Phosphorylated STAT3 dimerizes and translocates to the nucleus, where it induces transcription of target genes, such as Bcl-xL, Bcl-2, and survivin, which are critical for promoting neuronal survival [66]. Interestingly, the use of high doses of taxifolin (100 μM) increases STAT3 expression in brain cells by seven times [10], suppressing apoptosis, although in our study, the effect of 100 μM TAX was found to be comparable to that of 3 μg/mL SeNPs (Figure 7). Bcl-2 and Bcl-xL have well-described antiapoptotic properties [67,68,69], and we revealed that Se–TAX was most effective in enhancing the expression of Bcl-2.

The anti-inflammatory effect of taxifolin has also been shown: its administration (30 mg/kg/day) leads to a decrease in the expression of TNFα, NOS, IL-1β, Caspase-3, and Bax, which occurs simultaneously with an increase in the level of Bcl-2 in the hippocampus and cerebral cortex of LPS-treated mice [70]. A similar anti-inflammatory effect was shown for selenium compounds and naked SeNPs. Selenium compounds also activate mitochondrial biogenesis and, as a consequence, the level of intracellular ATP and Ca^2+^ homeostasis and promote cell survival in the penumbra zone [20,71]. Other authors have found in the model of epilepsy that SeNPs not only suppress oxidative stress, but also suppress the inflammation due to the inhibition of NF-κB [1]. Under current experimental conditions, using the same concentrations of compounds, the anti-inflammatory action and inhibition of TNFα was shown for “naked” SeNPs and the Se–TAX nanocomplex.

Note that there are only few studies on the cytoprotective mechanisms of action of taxifolin in the form of nanocomplexes with selenium, especially in complex neuroglial networks during ischemia and reoxygenation. The taxifolin nanocomplex with nanoselenium—P80-Que@Se nanocomposites (P80-Que@Se NCs)—was shown to suppress the aggregation of Aβ 1–42, to attenuate oxidative stress, and to hold promise for the treatment of Alzheimer’s disease [72]. It is shown that animals treated with taxifolin-loaded nanoemulsion had a significant improvement in the beam walking and open field tests. Additionally, taxifolin-loaded nanoemulsion was able to reduce the size of the hematoma, preserving the activity of glutathione S-transferase (GST) and increasing GSH content and the total antioxidant capacity [73]. Obviously, the use of taxifolin in the form of a nanocomplex with selenium shows an extremely high neuroprotective effect with the absence of significant side effects. As current studies have shown, the Se–TAX nanocomplex combines the best characteristics of an antioxidant, taxifolin, and its carrier, nanoselenium, which has an antiapoptotic effect. Se–TAX has a complex effect on brain cells, activating both antioxidant and antiapoptotic signal transduction pathways, involving the regulation of Ca^2+^ cell dynamics and the expression of genes encoding proteins responsible for cell survival. 

## 5. Conclusions

The developed selenium–taxifolin nanocomplex, created on the basis of selenium nanoparticles with a size of 100 nm, showed high cytoprotective efficiency in the cortical cells during ischemia/reoxygenation and the action of exogenous H_2_O_2_. The nanocomplex has high antioxidant properties and antiapoptotic and anti-inflammatory effects, increasing the level of expression of protective proteins and suppressing the expression of damaging ones. In general, the effectiveness of the studied agents in ischemia/reoxygenation can be arranged in descending order when using similar concentrations: Se–TAX > SeNPs > TAX. It should be said that the use of a nanocomplex based on selenium nanoparticles doped with taxifolin is a promising neuroprotective approach for brain ischemia due to the low toxicity of both components of the nanocomplex. At the same time, the prospect of using the nanocomplex is provided by the effect of the selective delivery of effective doses of taxifolin with the help of taxifolin. The limitation of this nanocomplex should consider the need for additional in vivo experiments.

## Data Availability

The data presented in this study are available on request to the corresponding author.

## Supplementary Materials

The following supporting information can be downloaded at: https://www.mdpi.com/article/10.3390/pharmaceutics14112477/s1, Figure S1: The effect of 24 h incubation of cortical cells with 3 μg/mL of selenium nanoparticles (SeNPs), nanocomplex Se–TAX, and free taxifolin (3 µg/mL or 100 µg/mL). Double staining of cells with Hoechst 33342 (HO342) and propidium iodide (PI) merges HO342 with PI and bright-field microscopy (BF). Control—cells without OGD/R. OGD/R—induction of OGD (2 h) and reoxygenation (24 h) without pretreatment with selenium-containing agents or free taxifolin. Figure S2: The effect of 24 h incubation of cortical cells with 3 μg/mL of selenium nanoparticles (SeNPs), nanocomplex Se–TAX, and free taxifolin (TAX).
