# Supplementary material for: Cytoprotective Properties of a New Nanocomplex of Selenium with Taxifolin in the Cells of the Cerebral Cortex Exposed to Ischemia/Reoxygenation
Source: Pharmaceutics. 2022 Nov 16;14(11):2477. doi: 10.3390/pharmaceutics14112477 (PMC9697510; doi:10.3390/pharmaceutics14112477)
Supplement: Supplementary file 1 [file pharmaceutics-14-02477-s001.zip › pharmaceutics-1995952-supplementary.pdf]

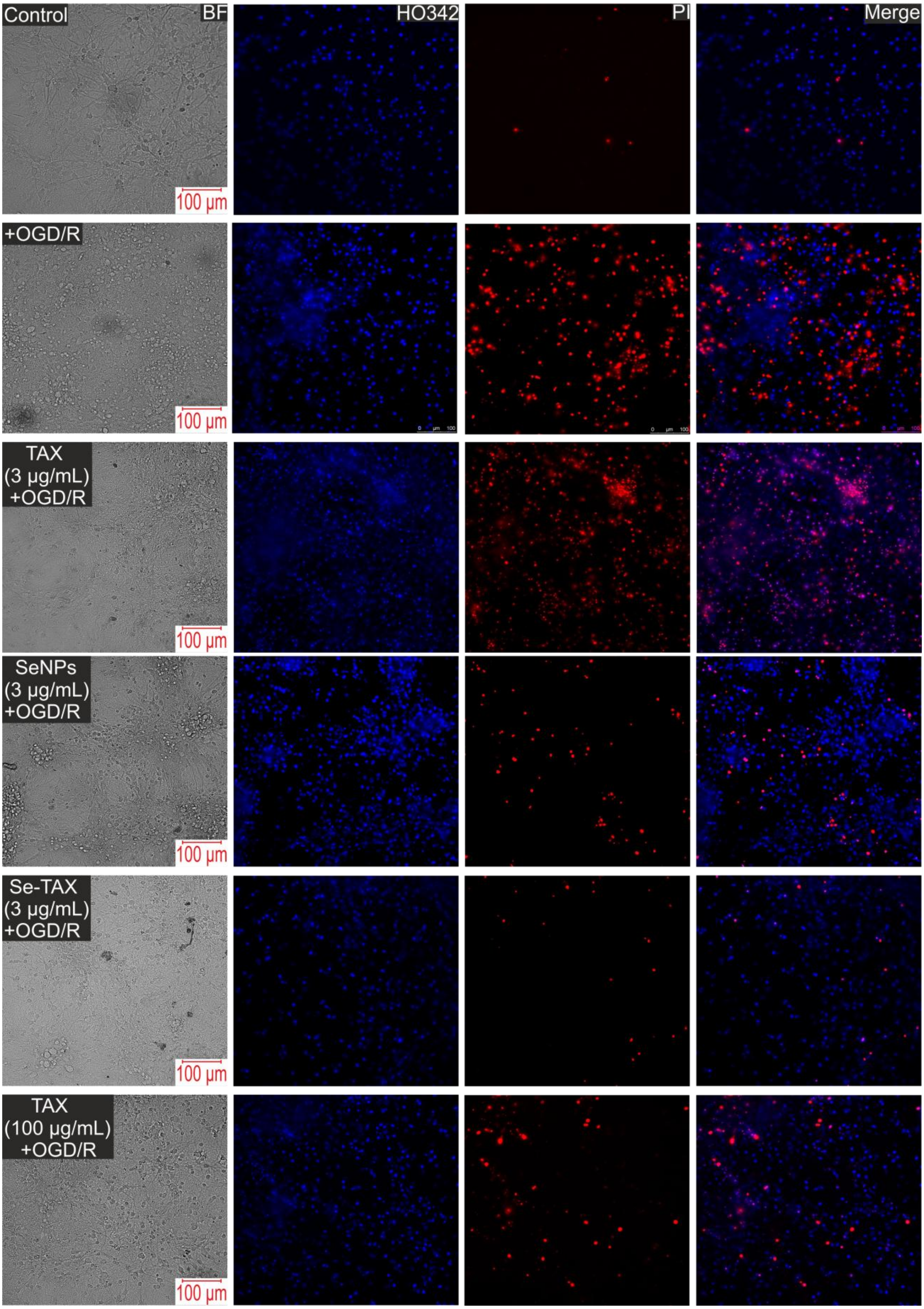

Supplementary Figure S1. The effect of 24-hour incubation of cortical cells with 3 µg/mL of selenium nanoparticles (SeNPs), nanocomplex Se-TAX and free taxifolin (3 µg/mL or 100 µg/mL). Double staining of cells with Hoechst 33342 (HO342), Propidium iodide (PI),

merge HO342 with PI and bright-field microscopy (BF). Control – cells without OGD/R. OGD/R – induction of OGD (2 h) and reoxygenation (24 h) without pre-treatment with selenium-containing agents or free taxifolin.

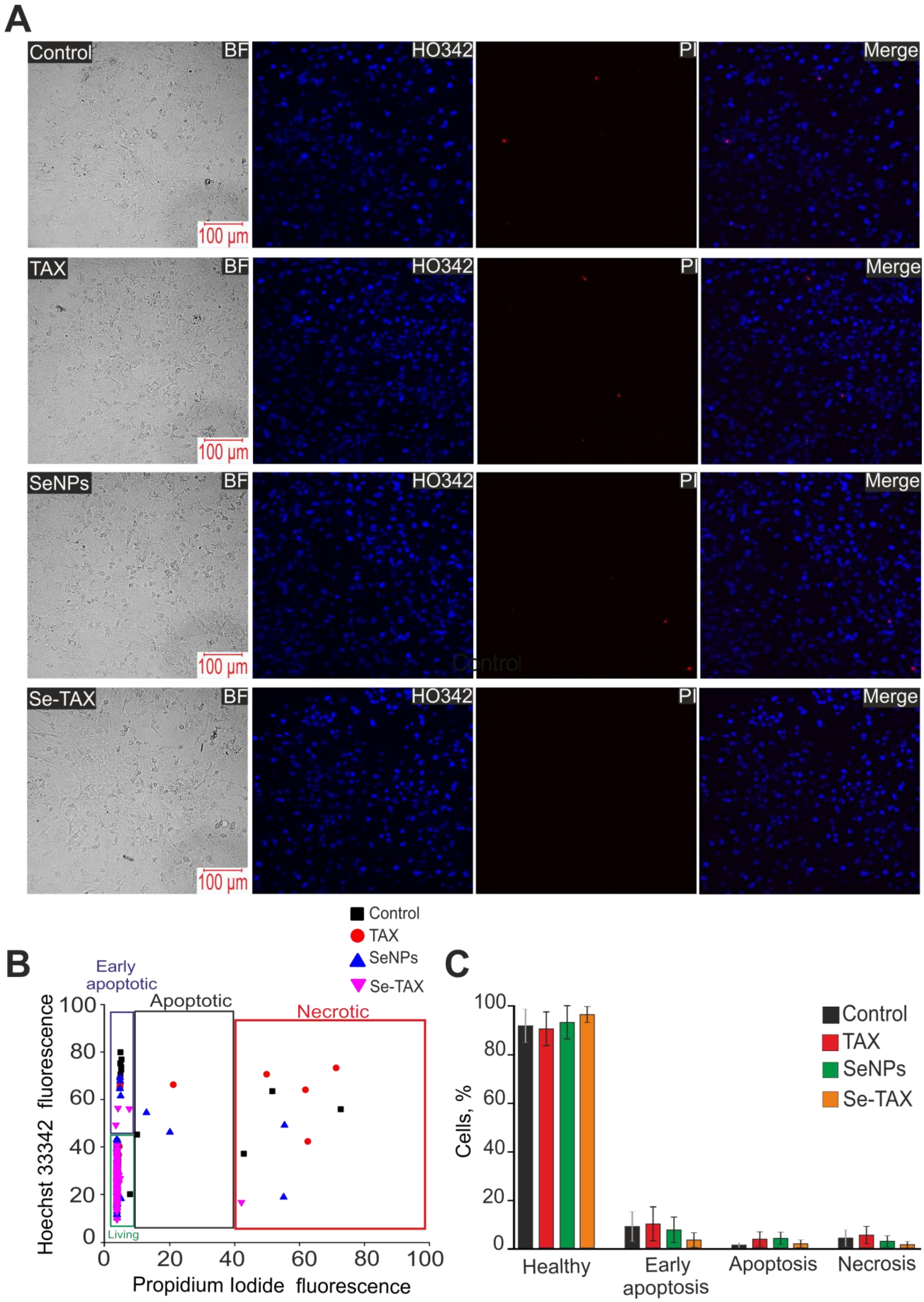

Supplementary Figure S2. The effect of 24-hour incubation of cortical cells with 3 μg/mL of selenium nanoparticles (SeNPs), nanocomplex Se-TAX and free taxifolin (TAX). (A) - Double staining of cells with Hoechst 33342 (HO342), Propidium iodide (PI), merge HO342 with PI and bright-field microscopy (BF). Control – cells without compounds. (B) - Cytogram demonstrating the viability of cortical primary cultured cells in Control (without compounds) and after 24-h pre-incubation with 3 μg/mL TAX, SeNPs or Se-TAX. X-axis – the intensity of PI fluorescence; Y-axis – the intensity of Hoechst 33342 fluorescence. Cells were stained with the probes 24 hours after the application of compounds. (C) - Effect of 24 hours pre-incubation with 3 μg/mL TAX, SeNPs or Se-TAX on the induction of necrosis and apoptosis. N cell cultures = 5; n cover slips with cells for each sample = 5. Pre-incubation of cells with the studied compounds does not cause reliable cell death.
